# Association between Alcohol Intake and Abdominal Obesity among the Korean Population

**DOI:** 10.4178/epih/e2010007

**Published:** 2010-05-19

**Authors:** Mikyung Ryu, Heejin Kimm, Jaeseong Jo, Sun Ju Lee, Sun Ha Jee

**Affiliations:** Department of Epidemiology and Health Promotion, Institute for Health Promotion, Graduate School of Public Health, Yonsei University, Seoul, Korea.

**Keywords:** Alcohol intake, Abdominal obesity

## Abstract

**OBJECTIVES:**

Although abdominal obesity has been reported to be highly related with alcohol intake, the results are still inconclusive. Therefore, this study was conducted to explore the association between alcohol and abdominal obesity among the Korean population.

**METHODS:**

This study included 8,603 participants (men: 5,195, women: 3,408) aged 30 to 87 who visited the health promotion centers in Seoul for routine health examinations from April, 2006 to June, 2007. Abdominal obesity was defined as WC ≥90 cm for men and ≥85 cm for women in accordance with the Korean Society for the Study of Obesity. For ever drinkers, total alcohol consumption in grams was classified into four groups (group 1, non-drinkers; group 2, 1-10 g of alcohol per day; group 3, 11-20 g of alcohol per day; and group 4, over 20 g of alcohol per day).

**RESULTS:**

The mean age of the study population was 45.4 yr old (men) and 45.3 yr (women). The average waist circumference was 85.3 cm in men and 75.3 cm in women. A high alcohol intake was associated with high waist circumference in both genders. In multivariate analysis, the group of men and women drinkers consuming >20 g in a day had a large waist circumference compared with men and women non-drinkers.

**CONCLUSION:**

This study showed that a high alcohol intake was related to high waist circumference. Such association remained independently even after adjustment for smoking, which is strongly related to abdominal obesity.

## INTRODUCTION

The increasing global prevalence of obesity has important health and economic consequences [1]. Obesity is a known risk factor for cardiovascular disease, ischemic heart disease, stroke and type 2 diabetes [2-4]. Because of insufficient physical exercise caused by economic expansions, fast-food diet and sedentary lifestyle, the prevalence of obesity has rapidly increased in Korea. According to KNHNES (Korea National Health & Nutrition Examination Survey) in 2007, people aged 20 or over are faced with a prevalence of obesity, defined as Body Mass Index (BMI) ≥25 kg/m^2^, of 31.8% (men 35.2%, women 28.3%). There has been an increase of around 1.2% compared with 2001 (30.6%: men 32.4% and women 29.4%) [5].

Recently, abdominal obesity has been acknowledged as more important than obesity generally. Both have been reported to be highly correlated with alcohol intake [6]. but there are some studies which show that regular alcohol intake is not involved in the development of abdominal obesity [7].

Several cross-sectional studies have evaluated the association between alcohol and abdominal obesity. but the results are still inconclusive [8]. Some found a positive association [9-11], whereas others found a negative or no association between alcohol intake and abdominal adiposity in both men and women [12-14]. Therefore, this study was conducted to explore the association between alcohol and abdominal obesity among the Korean population.

## METHODS

### Study population

This study included 9,995 Koreans (men: 5,824, women: 4,171) aged 30 to 87 who visited the health promotion centers in Seoul for routine health examinations from April, 2006 to June, 2007. Of the 9,995 participants, we excluded 223 who were younger than 30 and 1,105 with missing information on alcohol consumption and metabolic syndrome components. In addition, 64 participants were excluded because they had a past history of cardiovascular disease. Therefore, a total of 8,603 participants (men: 5,195, women: 3,408) were included in this study. Recruitment of volunteers only took place after written informed consent had been obtained. The Institutional Review Board of Human Research of Yonsei University approved this study.

### Anthropometric measurements and blood testing

Waist circumference (WC) was measured midway between the lower rib and iliac crest with a measuring tape. In cases of difficulty, WC was measured 3 cm above the navel. The participants wore underwear and exposed their waists while WC was measured by well-trained employees. Participants' weight and height were also measured while they were wearing light clothing. BMI was calculated as weight (kg) divided by the square of height (m^2^). Both systolic and diastolic blood pressure were measured after a fifteen minute rest. For the clinical chemistry assay, serum was separated from peripheral venous blood samples that were obtained from each participant after twelve hours of fasting, and was then stored at -80℃. Metabolic syndrome components, such as fasting blood glucose, total cholesterol, triglyceride, and high density lipoprotein cholesterol (HDL-C) were measured with a Hitachi-7600 analyzer (Hitachi Ltd., Tokyo, Japan).

### Self-questionnaire

Each participant was interviewed using a structured questionnaire to collect smoking habits, alcohol consumption and physical activity as well as other demographic characteristics such as age, gender, income and marriage status. Income (<3,000,000 or ≥3,000,000 KRW), marital status (single or married), and physical activity (yes or no) were divided into two groups. Cigarette smoking was classified as never smoker, ex-smoker and current smoker. Alcohol consumption was divided into two groups-non-drinker and always drinker. For the latter, we calculated alcohol consumption using the following formula: alcohol consumption (gram per day)=(No. of alcohol intake per week)×13 gram (amount of alcohol in gram per cup) / 7 days

### Definition of abdominal obesity

Abdominal obesity was defined as WC ≥90 cm for men and ≥85 cm for women in accordance with Korean Society for the Study of Obesity criteria [15]. Obesity was defined as BMI ≥25 kg/m^2^ according to the WHO definition [16].

### Statistical analysis

For evaluation of the general characteristics among the study population, means and standard deviations (SD) were calculated and frequency of smoking and alcohol consumption was determined. Total alcohol consumption in grams was classified into four groups (group 1, non-drinkers; group 2, 1-10 g of alcohol per day; group 3, 11-20 g of alcohol per day; and group 4, over 20 g of alcohol per day). Logistic regression analysis was performed to determine the association between total alcohol consumption and WC after adjustment for age, smoking habits, physical activity, education levels, income, and marriage status. All analyses were conducted with SAS statistical software, version 9.1 (SAS Institute Inc, Cary, NC, USA). Statistical significance was assumed to be p<0.05 when required.

## RESULTS

The prevalence of obesity was found to be 27.7% (men: 27.2%, women: 28.4%) by NCEP-ATP III whereas it was 21.7% (men: 27.2%, women: 13.3%) according to the definition of obesity for Koreans [15].

Table 1 shows general characteristics of the study population. The mean age of the study population was 45.4 yr for men and 45.3 yr for women. The average WC was 85.3 cm in men and 75.3 cm in women. The mean body mass index among men was 24.6 kg/m^2^, whereas it was 22.7 kg/m^2^ among women. About 88.7% of men were drinkers who drank 16.3 g in a day, whereas 44.5% of women were drinkers who drank 2.5 g in a day.

Table 2 displays changes in general characteristics of men according to the amount of alcohol consumed in a day. Compared with non-drinkers, current drinkers had significantly increased diastolic blood pressure (DBP), fasting serum glucose (FSG), total cholesterol (TC) and gamma glutamyltransferase (GGT) with increasing consumption of alcohol use. About 47.4% of men drinkers consuming >20 g in a day were aged between 40 and 49.

Table 3 displays changes in general characteristics of women according to the amount of alcohol consumed in a day. Compared with non-drinkers, current drinkers had significantly increased levels of FSG, high density lipoprotein cholesterol (HDL-C) and GGT with increasing amount of alcohol use. About 45.5% of women drinkers consuming > 20 g in a day were aged between 30 and 39.

In the correlation between obesity-related variables and alcohol intake for both genders, after adjustment for age, WC, SBP, DBP and FSG were significantly positively correlated with alcohol intake. In particular, men had positive correlations with additional obesity-related variables such as BMI, triglyceride and TC. Additionally, a high alcohol intake was associated with high waist circumference in men (data not shown). After adjustment for age, smoking status, physical activity, income and marriage status, compared with non-drinkers, the group of current drinkers consuming > 20 g in a day had significant associations with central obesity for men and women (Table 4).

## DISCUSSION

Waist circumference may have certain advantages over other measurements of adiposity in predicting risk of obesity-related diseases [17]. For example, the specificity and sensitivity of waist circumference is higher in terms of predicting hypertension, dyslipidemia and diabetes compared with body mass index, and waist circumference was consistently more accurate [18].

In this study, waist circumference was measured among 8,603 Korean adults (men: 5,195, women: 3,408) to assess the relationship with alcohol intake. After adjustment for age, smoking, physical activity, income and marriage status, men and women with a high alcohol intake were positively correlated with high waist circumference.

In previous studies on the relationship between alcohol and high waist circumference, some found a positive association [9, 10, 19-21], whereas others found a negative or no association in both men and women [12-14]. A Korean study in 2006 revealed an increase in the risk of obesity with increasing amount of alcohol consumption in both genders [22]. This present study determined a positive association between alcohol and high waist circumference in both genders. A study of Italian women, however, showed that risk of obesity increased with increasing amount of alcohol consumption [23]. Additionally, alcohol was not associated with increasing waist circumference among 16,587 men participating in the Health Professionals Follow-up Study [24]. A Danish study also reported an inverse association of waist circumference (measured ten years after the baseline) with total drinks of wine consumed per week in both genders. Those who consumed one to seven drinks per week had smallest waist circumference [25]. In contrast to these studies, two other studies, a Finnish study of more than 12,000 adults and a British study of 7,608 men have found that heavy alcohol drinking is associated with increased risk of obesity [26-28].

Probable mechanisms have been suggested in previous studies. Alcoholic beverages are energy dense and may not be substituting for food but rather add to the total daily energy intake [29]. Inhibition of fat oxidation might occur as a consequence of the antilipolytic properties of metabolites from alcohol degradation [30]. These features could potentially promote fat storage and hence promote an increased risk of developing obesity [31].

The strength of our study was that it is a large population-based study consisting of 8,600 Korean men and women. It does, however, have several limitations. The major limitation lies in its cross-sectional nature which precludes causality. A selection bias may exist, as most participants included in this study would be health conscious. Therefore, this finding cannot be applied to the general population. Another limitation may be that waist circumference was measured only once. Finally, the most significant factors, such as dietary habits, affecting abdominal obesity were not adjusted in this study.

In conclusion, this large population-based study showed that a high alcohol intake was related to high waist circumference. Such association remained independently even after adjustment for smoking, which is highly related to abdominal obesity. Improvement of alcohol intake may help in treating abdominal obesity. Therefore, further studies should be performed to confirm the association between alcohol intake and abdominal obesity.

## Figures and Tables

**Table 1:**
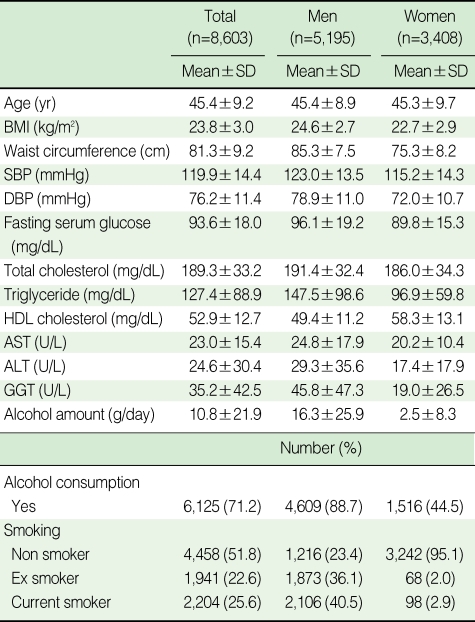
General characteristics of study population

SD, Standard deviation; BMI, Body Mass Index; SBP, Systolic blood pressure; DBP, Diastolic blood pressure; HDL-cholesterol, High density lipoprotein cholesterol; AST, Aspartate aminotransferase; ALT, Alanine aminotransferase; GGT, Gamma glutamyltransferase.

**Table 2:**
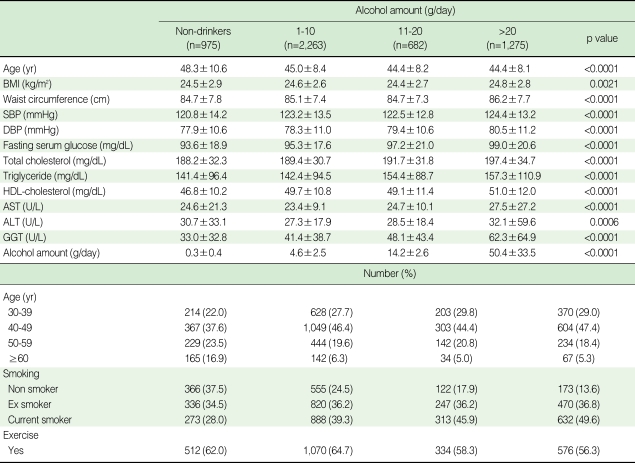
Baseline characteristics of study population according to alcohol amount in men (n=5,195)

SD, Standard deviation; BMI, Body Mass Index; SBP, Systolic blood pressure; DBP, Diastolic blood pressure; HDL-cholesterol, High density lipoprotein cholesterol; AST, Aspartate aminotransferase; ALT, Alanine aminotransferase; GGT, Gamma glutamyltransferase.

**Table 3:**
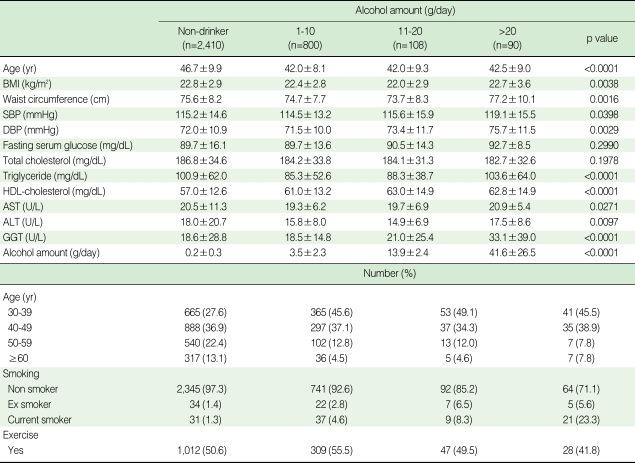
Baseline characteristics of study population according to alcohol amount in women (n=3,408)

SD, Standard deviation; BMI, Body Mass Index; SBP, Systolic blood pressure; DBP, Diastolic blood pressure; HDL-cholesterol, High density lipoprotein cholesterol; AST, Aspartate aminotransferase; ALT, Alanine aminotransferase; GGT, Gamma glutamyltransferase.

**Table 4:**
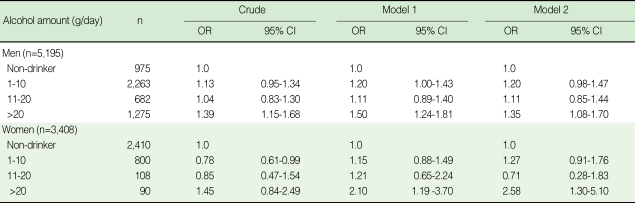
Odds ratios (95% CI) of central obesity according to levels of alcohol consumption

Model 1, adjusted age; Model 2, adjusted age, smoking status, exercise, income, marriage. Central obesity: men (90=<waist), women (85=<waist).
